# Leaf color change and photosystem function evaluation under heat treatment revealed the stress resistance variation between *Loropetalum chinense* and *L. chinense* var. *rubrum*

**DOI:** 10.7717/peerj.14834

**Published:** 2023-02-13

**Authors:** Wenqi Cai, Damao Zhang, Xia Zhang, Qianru Chen, Yang Liu, Ling Lin, Lili Xiang, Yujie Yang, Lu Xu, Xiaoying Yu, Yanlin Li

**Affiliations:** 1 College of Horticulture, Hunan Agricultural University, Changsha, Hunan Province, China; 2 Ministry of Education, Engineering Research Center for Horticultural Crop Germplasm Creation and New Variety Breeding, Changsha, Hunan Province, Chian; 3 Hunan Mid-Subtropical Quality Plant Breeding and Utilization Engineering Technology Research Center, Changsha, Hunan Province, Chian; 4 School of Economics, Hunan Agricultural University School of Economics, Changsha, Hunan Province, China; 5 Kunpeng Institute of Modern Agriculture China, Foshan, Guangdong Province, China

**Keywords:** Heat stress, Leaf color, Photosystem function, *Loropetalum chinense* var. *rubrum*

## Abstract

This research mainly focused on the leaf color change and photosystem function differentiation between *Loropetalum chinense* and its variety *L. chinense* var. *rubrum* under heat stress, which were tightly concerned about their ornamental traits and growth. *L. chinense* ‘Xiangnong Xiangyun’ (X) and *L. chinense* var. *rubrum* ‘Xiangnong Fendai’ (F) and *L. chinense* var. *rubrum* ‘Hei Zhenzhu’ (H) were chosen to be experimented on to investigate whether leaf color morphology and pigment composition could influence the adaptability of plants to high temperature in order to select foliage plants which posses stable leaf color and better adaptability for hot regions. The plants were cultured in hot environment (40 °C/33 °C, day/night) and normal environment (25 °C/18 °C, day/night). Phenotype and anatomic observation of three cultivars were made and leaf color indices and pigment contents were measured. During the experiment, H and F gradually turned green, total anthocyanins contents significantly decreased in them, however, chlorophyll b contents increased in all three cultivars. In addition, the initial fluorescence (F_o_) decreased in X, while increased in H and F. For the maximum fluorescence (F_m_) and maximum photochemical efficiency of PSII (F_v_/F_m_), they only increased in H and decreased in both F and X. The non-photochemical chlorophyll fluorescence quenching (NPQ) also increased in H and decreased in F. For X, it increased at first then gradually decreased. The coefficient of photochemical quenching all increased at first then gradually decreased. Correlation analysis between showed that there was relatively strong connection between anthocyanins, flavonoids and chlorophyll fluorescence parameters, especially NPQ, proved anthocyanins and flavonoids might not only involved in enriching leaf color, but also interfered with the protection of photosystem. Generally speaking, we found higher anthocyanin and flavonoids content level not only dramatically enriched the leaf color of *L. chinense* var. *rubrum* cultivars, but also offered more potential antioxidant to keep their normal growth when encountered heat stress.

## Introduction

Global warming has caused more and more negative effects on growth and reproduction process of plants (Haque, Hossain & Sushmoy, 2019). As continuous heat stress influenced not only normal growth of plants, but also ornamental traits of them. For example, heat stress could restrain development of capitulum during inflorescence meristem formation and floret growth in several *Chrysanthemum* species (Nakano et al., 2013). It also reduced the number of flowers and shortened florescence in *Rosa chinensis* (Hu et al., 2008). As for foliage plants, leaf traits are important factors when they were used in landscape, whether leaves were healthy was highly connected with normal growth and morphological traits of them (Zhu, Zhang & Qi, 2010).

Environmental factors, such as Light, moisture and temperature would all influence leaf traits. Among them, temperature, especially high temperature showed extremely significant effects on leaf morphology and physiology. Leaves usually wilted and became yellow when suffered from heat stress (Ou, Cao & Zheng, 2008). When chronically exposed to extremely high temperature, the outlook of plants would be continuously deteriorated, causing leaf discoloration, loss of turgor and decrease of chlorophyll content (Zhou & Liang, 2021). These negative changes of leaves were mainly due to intrinsic components change and structure failure (Shareef, Abdi & Fahad, 2020; Zandalinas et al., 2016). In this process, leaf pigment contents were deeply involved. They did not only determine color of leaves, but also took part in the resistance to heat stress, especially in the photosystem. Chlorophyll, carotenoids, and anthocyanins are main reason for leaf color presentation (Archetti, 2000). As reported, the pigment contents change happened in many plants when they encountered heat stress. Chlorophyll content of Japanese Maple was positively correlated with temperature, while the anthocyanin content was negatively correlated (Chen et al., 2010). However, there was also research showed that plants usually showed a trend of decrease in chlorophyll under heat stress, thus turned leaves into yellow and influenced the absorption and utilization of light energy of leaves (Dutta, Mohanty & Tripathy, 2009). Chlorophyll fluorescence technique was usually used to detect whether abiotic and biotic stress did harm to the photosystem, as it broader indicator of how plants respond to environmental change (Murchie & Lawson, 2013). In *Chenopodium album*, it was proved that chlorophyll contents was highly connected with PSIIfunction, especially the non-photochemical chlorophyll fluorescence quenching (Tsujimoto & Hikosaka, 2021). Anthocyanin could protect the photosystem by masking the chlorophyll containing organelles or absorbing at the same wavelength as chlorophyll b to offer auxiliary function during senescence (Hoch, Zeldin & McCown, 2001; Pietrini, Iannelli & Massacci, 2002). There has been some research about leaf pigment contents and their response to high temperature, yet photosystem function, which was tightly concerned with the growth of plants under elevated temperature was less discussed (Mishra et al., 2020). We want to analyze whether leaf pigment contents were interfered in the process of photosystem resistance to high temperature, and then offer reference for selecting ornamental plants with higher adaptability for hot regions.

*Loropetalum chinense* and its variety *Loropetalum chinense* var. *rubrum* were very important ornamental and medicinal plants in China (Li et al., 2008). They were widely used in landscape for their unique ornamental traits and being easy to manage (Li et al., 2008). As foliage plants, heat stress would do harm to their appearance, especially their leaf color, which has caused severe problem for their popularization (Huang et al., 2017; Yuan, Jia & Duan, 2010). Thus, the decisive factors to keep normal growth and leaf color traits under heat treatment need to be detected for the usage of *L. chinense* and *L. chinense rubrum* in hot regions.

Pigment contents and composition not only gave *L. chinense* and *L. chinense rubrum* abundant leaf color, but also let their photosystem function presented significant variation in shade-tolerance, capacity of CO_2_ utilization and light energy, *etc*. (Dong et al., 2016). Whether the photosystem function would show different change under heat stress among *L. chinense* and *L. chinense rubrum* cultivars with different color of leaves remains unclear, similar studies have been reported in other species. In *Begonia semperflorens*, the cultivar with colored leaves showed higher NPQ value and less changed Fv/Fm value and Y (II) value when under heat stress, turned out that it was more heat-tolerant than the cultivar with green leaves (Chang, 2013). Researches on *Cotinus coggygria* and *Cotinus coggygria* var. *purpurens* also showed that photosystem of the red-leaf variety showed more resistance to heat stress (Qi, 2008). Thus, we were about to research ornamental traits and adaptability of *L. chinense* and *L. chinense* var. *rubrum* under high temperature to see whether the variation of their leaf color would grant them different heat resistance ability. Through investigating the difference of response to heat stress between *L. chinense* and its variety *L. chinense* var. *rubrum*, we revealed that contents of anthocyanin and flavonoids would influence the resistance of *L. chinense* var. *rubrum* cultivars to heat stress, which might offer them more adaptability in hot regions.

## Material and Methods

### Plant material and growing condition

Two-year old cuttings of *Loropetalum chinense* ‘Xiangnong Xiangyun’ (X), *Loropetalum chinense* var. *rubrum* ‘Hei Zhenzhu’ (H) and *Loropetalum chinense* var. *rubrum* ‘Xiangnong Fendai’ (F) were planted in 7.57 L pots. Then these plants were placed in an artificial climate box. The temperature was set as 25 ° C for the day (14 h)and 18 ° C for the night (10 h) and proper moisture and fertilization were offered to keep the plants healthily growth, the organic matter content of the soil was 1.07%, total nitrogen content was 0.12%, and available potassium content was 54.64 mg/kg. The light was LED light and the PPFD value was set as 30,000 lx, the relative humidity was set as 90%. More details were listed in Table 1. The mature functional leaves of them were used for photosynthetic analysis and phenotypic measurement. Under proper environment, the mature leaves of H were deep purple and black (RHS BALCK GROUP 202A), while those of F were reddish brown (RHS GREYED-ORANGE GROUP 166A), and those in X were green (RHS GREEN GROUP 141A).

**Table 1:** Environmental factors set in the artificial climate box. The table presents environment factors for experimental group and control group, the temperature units were °C, the photoperiod was 14 h of light and 10 h of dark. The PPFT units was lx, the relative humidity was 90%.

Treatment	Day temperature (°C)	Night temperature (°C)	Illumination time (hours)	Dark time (hours)	Light intensity (lx)	Humidity (%)
Heat group	40	33	14	10	30,000	90
Control group	25	18	14	10	30,000	90

### Heat treatment

Six pots of each cultivar were exposed to an environment of 40 ° C for the light (14 h) and 33 ° C for the night (10 h). The relative humidity was set as 90%. The control group was set as same as the experimental group despite its temperature was set as 25 ° C for the day (14 h) and 18 ° C for the night (10 h).

### Color evaluation

The Royal Horticultural Society Color Chart (RHSCC) was used to evaluate the leaf color change of each cultivar. Five healthy newly matured leaves were picked to compare with the standard color. Furthermore, YS 3020 spectrophotometer (3nh, China) was used to measure the color indices L^∗^, a ^∗^ and b^∗^.

### Pigment contents

Photosynthetic pigment including chlorophyll a, chlorophyll b and carotenoids, anthocyanins and flavonoids of three cultivars were measured. Photosynthetic pigment was measured by the means below: 0.2 g of fresh leaf samples were cleaned with deionized water and dried with lens paper, then were cut into even pieces, the pieces were soaked into 10 ml of 95% alcohol for 24 h of dark extraction, the absorbance at 470 nm, 649 nm and 665 nm were measured using a ultraviolet spectrophotometer (Li, 2020). Anthocyanin contents were measured using pH differential method (Chen et al., 2021), fresh samples were frozen in liquid nitrogen and grilled into powder to digest in 0.5% of methanol hydrochloride for twenty four hours, then the extract was centrifuged, the supernatant was added into buffer A (0.4M potassium chloride solution, the pH was modified to 1.0 using hydrochloric acid) and buffer B (1.2M citric acid solution, the pH value was modified to 4.5 using disodium hydrogen phosphate), respectively. The absorbance at 510 nm and 700 nm were measured for the calculation of anthocyanin contents. Flavonoids contents were detected with the reference to the method of Heimler. 0.1 g of fresh sample was ground in to powder using liquid nitrogen and mixed with 95% of methanol, after 24 h of dark extraction, the supernatant was diluted, 0.5 ml of the solution was then mixed with 0.2 mL of 5% NaNO_2_, 0.3 mL of 10% AlCl_3_ and one mL of 1 mol L^−1^ NaOH in turn, deionized water was used to make a constant volume of 3.5 mL, fully mixed all the solution and let them stand for 10 min, and finally measure the light absorption value at the wavelength of 510 nm, the catechin standard solution with a concentration range of 50 mg/L–250 mg/L was used to establish the standard curve and measured the relative content of flavonoids in the sample (Heimler et al., 2005). Each kind of pigment contents were detected before heat treatment and on the first day, the third day, the fifth day and the seventh day using methods mentioned above.

### Photosynthetic function and soluble sugar contents

The net photosynthetic rate and stomatal conductance were detected by LI-6400 Portable Photosynthesis System (LI-COR Biosciences, Lincoln, NE, USA) Leaves detected were exposed to strong light for ten minutes to ensure adequately activated. The soluble sugar contents were measured by anthrone colorimetry (Yue et al., 2022). 0.1 g of fresh sample were grounded into powder using liquid nitrogen, then mixed with 1 ml of ultrapure water. The mixture was heat-treated in the boiling water bath for 20 min and then cooled to the room temperature. After this, the mixture was centrifuged and the supernatant of it was gathered. 1 ml of supernatant was diluted to 10 ml, after all this, 400 µl of the solution was added with 400 µl of ultrapure water, 200 µl of anthrone solution (1 g of analytical pure anthrone diluted in 50 ml of ethyl acetate) and 2 ml of concentrated sulfuric acid, respectively. The components were completely mixed and then heat-treated in the boiling water bath for 10 min and then cooled to the room temperature. The absorptance at 620 nm was measured using a ultraviolet spectrophotometer.

### Chlorophyll fluorescence parameters

Five newly matured leaves for each cultivar were selected to test. The leaf-clips were fixed on the front of the blade, then closed, and waited for 20 min, and the clips were produced by Hansatech, Britain. After 20 min of dark adaptation, a FluorPen FP 110 handheld fluorescent meter was connected to the clip, the chlorophyll initial fluorescence (*F*_O_), maximum fluorescence (*F*_m_) and effective quantum yield of photosystem II (Φ PSII) were acquired through ojip protocol. The non-photochemical quenching coefficient (NPQ) and photochemical fluorescence quenching coefficient (qP) were acquired through NPQ 3 protocol.

### Correlation analysis between pigment contents and chlorophyll fluorescence parameters

Three replicants for pigment contents and chlorophyll fluorescence parameters were selected to carry correlation analysis. The parameters were placed in the graph and the Pearson correlation coefficient between each parameter were calculated by SPSS.

### Statistical analysis and presentation

Three replicants for each parameter were selected. Analysis of variance (ANOVA) and multiple mean comparisons were made on the parameters using SPSS Statistics, and correlation analysis between leaf color and chlorophyll fluorescence parameters was made. Data were expressed as mean ± SD. Different letters indicate significant differences at *p* ≤ 0.05 based on Duncan’s new multiple range test. The graph were made by Origin and RStudio, the pictures were modified and put together by Adobe Photoshop and Adobe Illustrator.

## Results

### Phenotypic observation on leaf color

**Figure 1:**
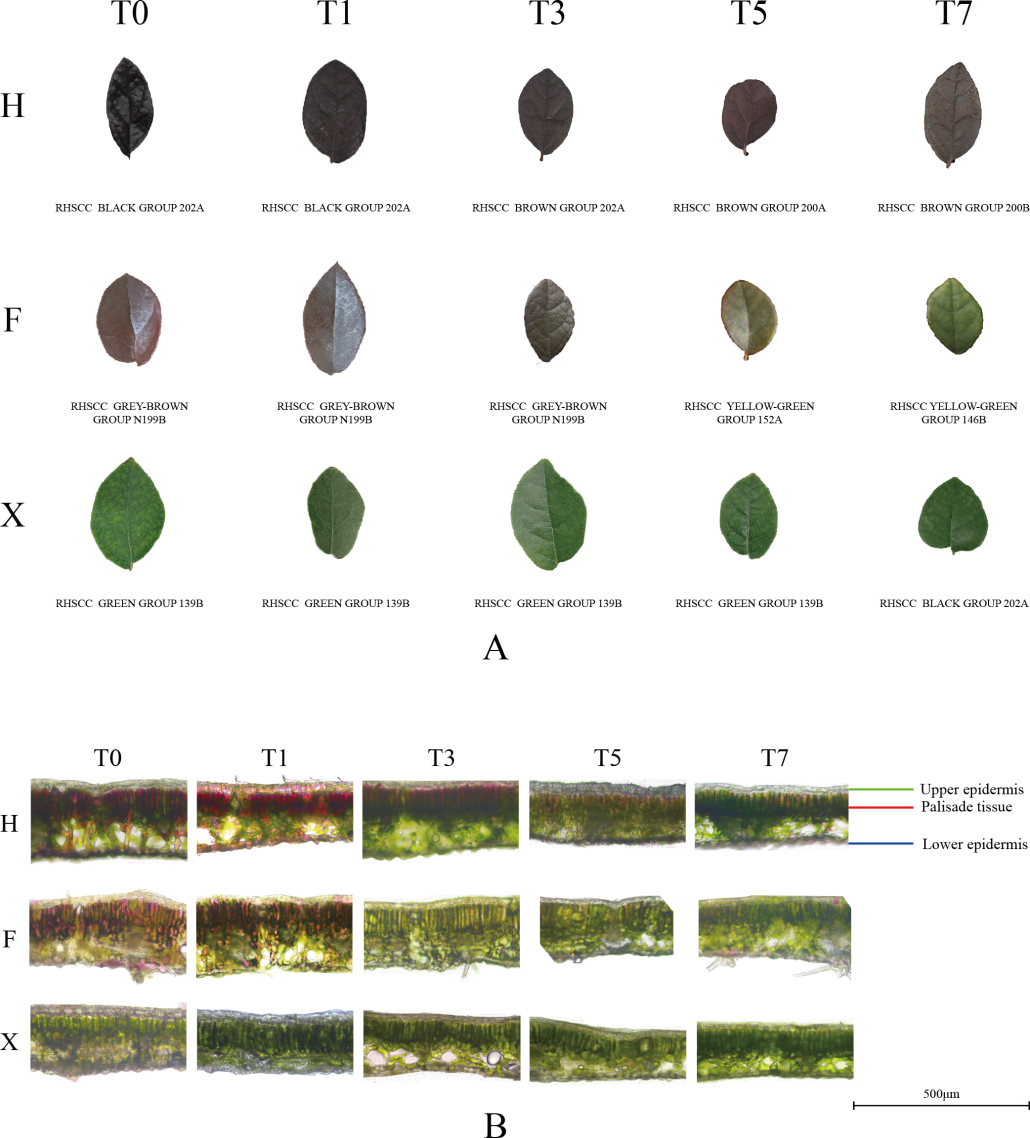
Phenotypic observation of leaf color change under heat stress. (A) Surface of leaves of three cultivars under heat stress. (B) Anatomic structure of leaves of three cultivars under heat treatment. (A) Leaf color of each stage was described by the RHSCC number to qualitatively evaluate the change trend when plants were under heat treatment. (B) In each picture, from top to bottom, the upper epidermis, palisade tissue, spongy tissue and lower epidermis of leaves were presented successively. Scale bars = 500 µm.

Qualitative description of three cultivars was listed in Table 2. As shown in Fig. 1A, the most apparent leaf color change showed in F, while in other cultivars, leaf color change seemed not being so visible. We then made an anatomical observation on the transection of the leaves, the consequence was showed in Fig. 1B. Before heat treatment, the palisade tissue of three H was dark purple, and several purple elements were also distributed in the epidermis and spongy tissue. Although there was not obvious color change on the leaf surface of H, it was observed that the palisade tissue and epidermis of H were both turning green from dark purple. The transection of F was mainly pink and green, while pink parts spread on the epidermis and palisade tissue. Under heat treatment, the pink parts in F leaves rapidly turned to green, on the third day of heat treatment, these parts almost vanished completely. The surface and the transection of X did not show significant change in eyes, indicated that its leaf color might be less influenced by heat stress.

**Figure 2:**
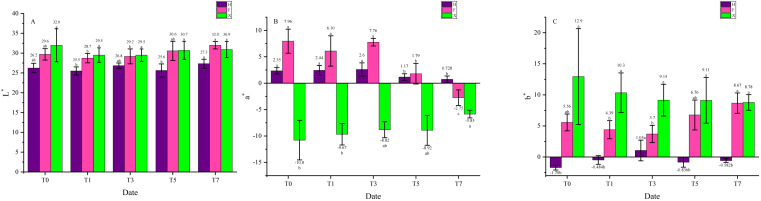
Color indices change under heat treatment. (A) Parameter L^*^. (B) Parameter a^*^. (C) Parameter b^*^. The color indices were used to quantitatively describe the leaf color. L^*^ presents Lightness, the number is bigger, the color is closer to white. a* presents red and green, the number is bigger, the color is closer to red, whereas to green. b^*^ presents yellow and blue, the number is bigger, the color is closer to yellow, whereas to blue. The purple bars indicate H, while pink bars indicate F, and green bars indicate X. Data were expressed as mean ± SD. Different letters indicate significant differences at *p* ≤ 0.05 based on Duncan’s new multiple range test.

The color parameters were listed and analyzed in Fig. 2 to quantitatively emphasis the leaf color change under heat stress. The parameter L ^∗^ did not change remarkably in all three cultivars, while in parameter a^∗^, H and F both showed a trend of decrease, and the variation was more significant in F compared with H. There was a diametrically opposed trend in a^∗^ of X, it gradually increased during the heat treatment. As for b ^∗^, this parameter did not change significantly in H, while it showed a trend of decrease in X. In F, it decreased first then increased to a higher level than the beginning. Interestingly, the obvious changes all happened between the third and the fifth day, which might be an important time slot in the leaf color change under heat stress. The results presented that leaf color of *L. chinense* var. *rubrum* turned green from red under heat treatment and this change was visible. Between two *L. chinense* var. *rubrum* cultivars, leaf color of F changed more significantly, indicated that this cultivar might be more sensitive to heat stress. Besides, the color indices of X also changed under heat stress and this change was hard to perceive, which could also influence the ornamental traits of it.

**Table 2:** Leaf color traits under control Group. (25 °C) The color was described by value of spectrophotometry and the number of the Royal Horticultural Society Color Chart.

Cultivar	Stage	L^*^	a^*^	b^*^	RHSCC
H	T0	26.24 ± 1.20^c^	2.35 ± 0.60^b^	−1.70 ± 0.40^c^	BLACK 202A
F	T0	29.69 ± 1.44^ab^	7.96 ± 2.28^a^	5.56 ± 1.36^ab^	GREY-BROWN N199B
X	T0	31.97 ± 4.17^a^	−10.79 ± 3.74^c^	12.94 ± 7.72^a^	GREEN 139B

**Notes.**

The parameter L^*^ indicates the lightness of leaves, the number is bigger, the color is closer to white. The parameter a^*^ indicates green and red, the number is bigger, the color is closer to red, otherwise it is closer to green. The parameter b^*^ indicates yellow and blue, the number is bigger, the color is closer to yellow, otherwise it is closer to blue. Different letters indicate significant differences at *p* ≤ 0.05 based on Duncan’s new multiple range test.

### Pigment contents change of leaves

**Figure 3:**
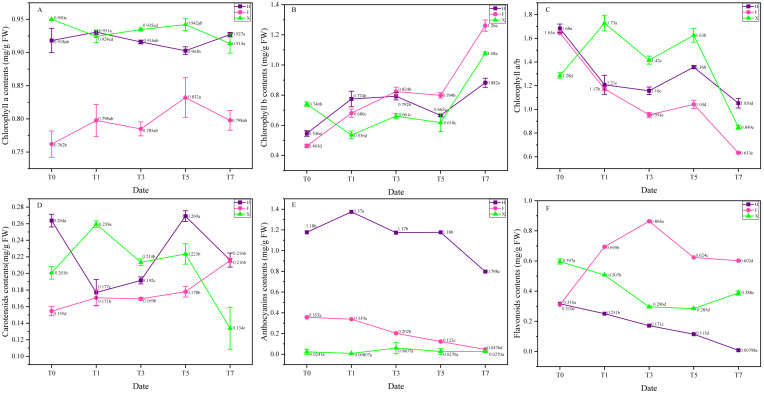
Pigment contents change under heat stress. (A) Chlorophyll a. (B) Chlorophyll b. (C) Chlorophyll a/b. (D) Anthocyanin. (E) Flavonoids. (F) Carotenoids. Each data point indicates the average performance of three runs of measured data. The deep purple line and point indicate the fluorescence of H, while the pink line and point indicate F, and green line and point indicate X. Data were expressed as mean ± SD. Different letters indicate significant differences at *p* ≤ 0.05 based on Duncan’s new multiple range test.

Chlorophyll, carotenoids, anthocyanin and flavonoids are decisive in the leaf color formation of *L. chinense* and *L. chinense* var. *rubrum* (Chen et al., 2021). The composition and proportion of these pigment contents was quite different among three cultivars (Table 3). Under heat treatment, the proportion of pigment contents showed significant change, thus triggered color change in the outlook of leaves, the contents change of pigment were presented in Fig. 3. There was no significant change in chlorophyll a among three cultivars, while chlorophyll b showed a palpable trend of increase in them (Figs. 3A and 3B), on the seventh day of heat treatment, contents of chlorophyll b increased 66% in H, 176% in F and 41% in X compared with the initial status. On contrary to chlorophyll b, anthocyanin showed a significant trend of decrease in H and F, on the seventh day of heat treatment, it decreased 32% and 88%, respectively. Flavonoids contents also changed dramatically under heat stress, In H and X, it sharply decreased after heat treatment on the first day, and continuously decreased. In F, however, its flavonoids contents abruptly increased at the first day and gradually increased. Carotenoids did not demonstrate obvious change trend in F and X, while in H, it was decreased at first and then increased again. Generally speaking, contents change of pigments triggered color change of leaves. Among them, anthocyanin and chlorophyll b played decisive role in the leaf color change of *L. chinense* var. *rubrum*.

**Table 3:** Leaf pigment contents at initial stage. This table presents the pigment contents level at initial stage, the pigment contents units were mg/g FW, the pigment contents were distracted from fresh samples of newly matured leaves. Data were expressed as mean ± SD. Different letters indicate significant differences at *p* ≤ 0.05 based on Duncan’s new multiple range test.

Cultivar	Chlorophyll a (mg/g FW)	Chlorophyll b (mg/g FW)	Chlorophyll a/b	Carotenoids (mg/g FW)	Anthocyanin (mg/g FW)	Flavonoids (mg/g FW)
H	0.91 ± 0.2^a^	0.54 ± 0.09^b^	1.69 ± 0.06^a^	0.27 ± 0.01^a^	1.18 ± 0.035^a^	0.32 ± 0.01^b^
F	0.76 ± 0.01^b^	0.46 ± 0.00^a^	1.65 ± 0.02^a^	0.15 ± 0.01^c^	0.35 ± 0.05^b^	0.31 ± 0.01^b^
X	0.95 ± 0.00^a^	0.77 ± 0.05^c^	1.23 ± 0.01^b^	0.20 ± 0.01^b^	0.05 ± 0.07^c^	0.57 ± 0.01^a^

### Effect of heat stress on net photosynthetic rate, stomatal conductance and soluble sugar contents

**Figure 4:**
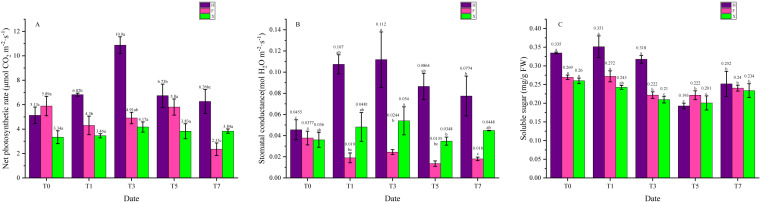
Net photosynthetic rate, stomatal conductance, and soluble sugar contents Change during Heat Treatment. (A) Net photosynthetic rate. (B) Stomatal conductance. (C) Soluble sugar contents. Each data point indicates the average performance of three runs of measured data. The deep purple bars indicate the fluorescence of H, while the pink bars indicate F, and green bars indicate X. Data were expressed as mean ± SD. Different letters indicate significant differences at *p* ≤ 0.05 based on Duncan’s new multiple range test.

As necessary catalyzer in photosynthesis, chlorophyll plays a central role in light absorption of photosynthesis and thus its content would influence the photosystem function. Besides, products of photosynthetic system were the basis for the stabilization of anthocyanins, hence leaf pigment contents were connected with photosynthetic system function, which is sensitive to high temperature. From Fig. 4A we can see that the net photosynthetic rate of H increased at first, then gradually decreased, while in F and X, it did not show significant change. Interestingly, the net photosynthetic rate in two *L. chinense* var. *rubrum* was higher than X when under 25 ° C. As for the stomatal conductance, its change trend was the same as the net photosynthetic in H. In X, however, it was also increased at first then decreased, for F, this parameter decreased on the first day of heat treatment and kept in a low level. Soluble sugar contents were relatively stable under heat treatment in F and X, yet it showed abrupt decrease between the third day and the fifth day in H.

### Chlorophyll fluorescence parameters

**Figure 5:**
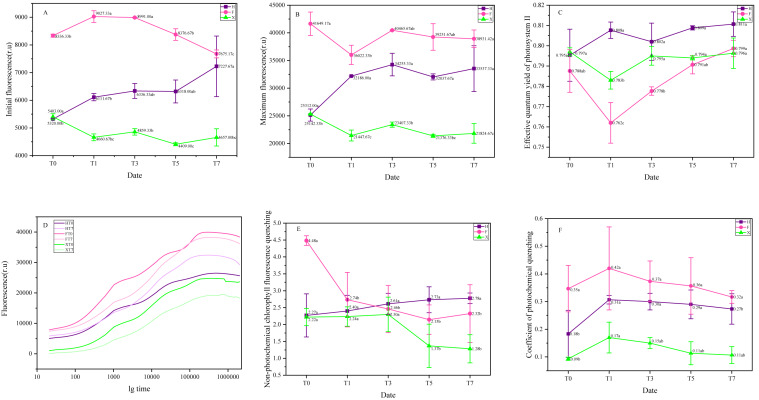
Representative chlorophyll fluorescence parameters and their change trend under heat treatment. (A) Initial fluorescence. (B) Maximal fluorescence. (C) Maximal quantum yield of PSII. (D) Non-photochemical chlorophyll fluorescence quenching. (E) Coefficient of photoc. Each data point indicates the average performance of three runs of measured data. The deep purple line and point indicate the fluorescence of H, while the pink line and point indicate F, and green line and point indicate X.

Among growth and reproduction of plants, photosystem played important role (Blankenship, 2010). As shown in Fig. 5, the highest value of *F*_o_ and *F*_m_ showed in F before heat treatment, while the effective quantum yield of photosystem II (*F*_v_/*F*_m_) of it was the lowest among three cultivars. For non-photochemical chlorophyll fluorescence quenching and coefficient of photochemical quenching, the highest values also appeared in F, turned out it had a better ability in photoprotection process and utilization of light energy.

Under heat treatment, *F*_o_ significantly increased in H and F on the first day, nevertheless, while X decreased at the same stage, during the heat treat period, only H showed continuous increase. For *F*_m_, F and X presented sharp decrease at the first day under heat treatment, while for H, it increased obviously with the stimulation of high temperature. The effective quantum yield of photosystem II ( *F*_v_/*F*_m_) decreased at the first day after heat treatment in F and X, then gradually increased in the next days, on the seventh day, it has become higher than initial stage in F, while in X, it could not rise fast as F despite it was keeping rising. In H, however, it increased at the first day of heat treatment and then got back to the normal level and gradually increased again.

Figure 5D presented the chlorophyll fluorescence dynamics curve of three cultivars, it was observed that the place of ojip curve of H showed a significant was higher after heat treated, turned out that plastid quinone reducing ability of it was significantly strengthened, in X, however, the place of its ojip curve was dramatically lower than initial stage, which indicated that it was not suitable for high temperature environment. The ojip curve of F also moved down, yet compared with X, the change range of F was not so significant, thus it was more resistant to heat stress.

The non-photochemical chlorophyll fluorescence quenching increased in H, which indicated that its photoprotection ability has been strengthened under heat treatment. In F, this parameter presented a sharp decrease at the first day and gradually decreased on the next days, turned out that this cultivar might be very sensitive to high temperature. There was no significant change in non-photochemical chlorophyll fluorescence quenching of X at early stage of heat treatment (T0-T3), but on the fifth day, it decreased dramatically. As for coefficient of photochemical quenching (QP), it was remarkably increased after heat treatment for one day, it was increased for 67% in H, 22% in F, and 83% in X, respectively. Then, it was slowly abated on the next days in all three cultivars.

According to the results, it seemed like F was more sensitive to heat stress as its change in fluorescence parameters was more significant and easier to be detected. H was suitable to hot environment, the chlorophyll fluorescence parameters all increased when it was cultured in relatively high temperature. The influence of high temperature on the PS II function was negative to X, making the ability of energy utilization and transformation weaker. Even the chlorophyll fluorescence parameters also decreased in F, however, the negative effects of high temperature were not presented as significant as they were presented in X, which indicated that F was also more tolerant to heat stress compared with X. Interestingly, two *L. chinense* var. *rubrum* cultivars both showed relatively higher resistance, which might indicate that their leaf color traits offered them stronger ability in heat-resistance.

### Correlation analysis between leaf pigment and photosystem in different colors of leaves

**Figure 6:**
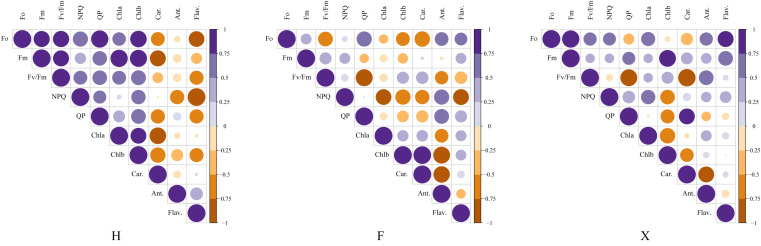
Correlation analysis between pigment contents and chlorophyll fluorescence parameters among three cultivars. This figure presented the pearson coefficients between chlorophyll parameters and pigment contents. The correlation analysis showed that the pearson coefficient between anthocyanin contents and NPQ value and flavonoids contents and NPQ value were higher in *L. chinense* var. *rubrum* cultivars,which indicated their higher photosystem stability under heat stress was partly due to the higher contents of non-photosynthetic pigment.

Figure 6 presented the consequence of correlation analysis between pigment contents and chlorophyll fluorescence parameters. In H, it was observed that the total chlorophyll contents were significantly correlated with the maximum fluorescence, while in F and X, this relation did not seem to be so close. There was extremely significant correlation between non-photochemical chlorophyll fluorescence quenching (NPQ) and flavonoids content in H, which might indicate that it was involved in the photoprotection mechanism, besides, although anthocyanin did not show such high correlation with NPQ as flavonoids, it was also highly correlated with it in H and F. As anthocyanins contents were higher in H and F, and the NPQ values were also higher in them, this probably indicated that pigment contents which not involved in photosynthesis could offer additional protection for photosystem.

## Discussion

### Effects of high temperature on the leaf traits of plants

Heat stress could influence the ornamental traits of plants by hindering their growth, making the leaves become smaller, lightening the leaf color or leading to premature senility (Liu et al., 2013). In most plants, chlorophyll is usually dominant in leaf color formation. It mainly absorbs blue and purple light meanwhile reflect green light thus presents green on the surface of leaves. Carotenoids offers yellow and orange for leaf color, compared with chlorophyll, it was relatively resistant to cold environment. Therefore, leaves of some species would become yellow in fall as chlorophyll was decomposed while carotenoids content was relatively stable (Su et al., 2015; Tang et al., 2020). As for coleus plants, anthocyanin plays important role in color formation, which offers various color for plants according to differentiation in pH value (Zhang et al., 2014). When anthocyanin contents were close to chlorophyll contents, they would complement and superimpose each other to make leaves present deep purple. In our research, the observation and detection on the phenotype and pigment contents of three cultivars of *L. chinense* and *L. chinense* var. *rubrum* were made to explain the leaf color change caused by high temperature. The consequence presented that high temperature made H and F leaves become greener than ever, and X did not show apparent change in leaf color. In the process of leaf color change, total anthocyanins contents and chlorophyll b contents played important role.

Anthocyanin decreased in H and F, as it was very sensitive to high temperature. several key genes of flavonoids and anthocyanin synthesis pathway expressed differently when suffered from heat stress. The regulation of temperature on genes in the anthocyanin biosynthesis pathway is mainly manifested in the upstream regulation of *PAL*, *CHS* and *CHI* genes and the downstream regulation of *DFR* and *ANS* genes (Huh et al., 2008). Besides, heat stress also decreased anthocyanins by turning them into colorless chalcone and methanol pseudobase (Sun et al., 2009). Between two *L. chinense* var. *rubrum* cultivars, F seemed to be more sensitive to heat as its leaf color changed faster and more under heat treatment, it might because the intramolecular co-pigmentation of anthocyanin contents was stronger in H than it (Figueiredo et al., 1996).

In general, we found that the leaf color change under heat treatment was due to the change of pigment contents, especially chlorophyll b and anthocyanins. Anthocyanins, which could offer leaves more red, gradually decomposed when in the hot environment, yet for H, it owned higher anthocyanins contents, thus gave it more ability to combine the anthocyanins molecules through hydrogen bond, dredge key and van der Waals to offer higher stability when suffered from heat stress (Teixeira et al., 2013). Chlorophyll b added more deep green for leaves, and comprehensively turned red leaves of H and X into green, however it also showed a trend of increase in X, this special mechanism might offer sufficient electron transport for the heat stability of PS II (Havaux & Tardy, 1997).

### Effects of high temperature on the photosystem function of plants

Photosystem was very sensitive to temperature (Allakhverdiev et al., 2008). Under heat stress, the photochemical efficiency of photosynthetic apparatus usually decreased, this was attributed to the structural change of chloroplast proteins D1 and D2, the decrease of enzyme activity, and the decrease of PS II activity caused by the damage of thylakoid membrane and oxygen producing complex. In addition, the degradation of pigments related to photosynthesis might also be one of the reasons for the decline of photosynthesis (Haldimann & Feller, 2004; Zhang et al., 2005).

In our study, H showed relatively higher adaptability under heat stress among three cultivars, as its net photosynthetic rate showed significant increased after treated, this was because higher temperature adequately activated the stoma of it, hence relieved the dominant stomatal Limitation (Pšidová et al., 2018). The maximal quantum yield of PSIIwas also the highest in H among three cultivars no matter whether it was under heat treatment or normal environment, which indicated it possessed the highest potential photo ability, this might also have made contributions to the higher anthocyanin synthesis in it since photo product-soluble sugar contents were also sufficiently supplied (Narayan & Venkataraman, 2002). Compared with other species, the photosystem function of our plant materials all showed adaptability under heat stress, the recovery of PS II function did not require much time, once they encountered current environmental change, they would swiftly moderate themselves to adapt unfavorable environment.

### Protection of leaf pigment content to photosystem function under high temperature

Leaf color was closely related to photosystem function. Existence of photosynthetic pigment was necessary condition of photosynthesis in higher plants (Gupta & Sinha, 2009). Besides, as another main chromogenic substance, anthocyanins need to be combined with photosynthate to form stable structure (Zhang et al., 2019). Correlation analysis between pigment contents and chlorophyll fluorescence were made to evaluate whether the changing leaf color could influence the photosystem function. According to the consequence of JIP-test, the biggest values of *F*_o_, *F*_i_, *F*_j_ and *F*_m_ all showed up in F under heat treatment, interestingly, its leaves were also the reddest among three cultivars on the basis of the spectrophotometer. Unlike two other cultivars, H showed relatively low chlorophyll fluorescence parameters among three cultivars, yet under heat treatment, *F*_o_, *F*_i,_ *F*_m_ and the maximal quantum yield of PSII all increased in it.

Anthocyanin contents seemed to offer more photoprotection ability for plants (Gould, McKelvie & Markham, 2002; Steyn et al., 2002; Tian et al., 2014). In our research, cultivars with higher anthocyanin contents showed higher non-photochemical chlorophyll fluorescence quenching (NPQ), it was partly because that anthocyanin enhanced the photoprotection ability by filtering or reflecting excess light energy (Hughes, Neufeld & Burkey, 2005; Kootstra, 1994; Li et al., 1993; Merzlyak et al., 2008). On the one hand, this mechanism could reduce quantum yield of photochemical reaction, on the other hand, it increased the proportion of energy dissipated by proton gradient and lutein cycle on both sides of thylakoid membrane (Zhou & Liang, 2021). Besides, the chlorophyll fluorescence parameters of cultivars with higher anthocyanin contents showed more stability under heat treatment, indicated that *L. chinense* var. *rubrum* which owns proper content of anthocyanins could not only improve their ornamental traits, but also increased their resistance to heat stress.

## Conclusion

Leaf color change, pigment contents change and photosystem function under heat treatment were evaluated in three cultivars. Photosystem stability of *Loropetalum chinense* var. *rubrum* cultivars showed more resistance to heat stress compared to *L. chinense*, turned out they might be more adaptative under hot environment, besides, cultivars with higher anthocyanin contents could keep their bright leaf color under high temperature, it indicated that they were suitable to be popularized in hot regions. The results showed that anthocyanin contents not only richened the ornamental traits of *Loropetalum chinense* var. *rubrum*, but also played important role in the resistance of plants to heat stress, and this might be an evolved mechanism in the adaptation to stress.

##  Supplemental Information

10.7717/peerj.14834/supp-1 Supplemental Information 1 Raw data of color parameters, pigment contents and other data

10.7717/peerj.14834/supp-2 Supplemental Information 2 Correlation analysis The analysis consequence for Fig. 6. The Pearson coefficients between chlorophyll fluorescence parameters and pigment contents were calculated.

10.7717/peerj.14834/supp-3 Supplemental Information 3 Fluorescence dataset The fluorescence parameters were analyzed using SPSS Statistics.

10.7717/peerj.14834/supp-4 Supplemental Information 4 Fluorescence parameters The fluorescence parameters were analyzed using SPSS Statistics.

10.7717/peerj.14834/supp-5 Supplemental Information 5 Pigment contents: analysis consequence Pigment contents were analyzed using SPSS Statistics.

10.7717/peerj.14834/supp-6 Supplemental Information 6 Net photosynthetic rate, conductance, soluble sugar Analyzed using SPSS Statistics.

10.7717/peerj.14834/supp-7 Supplemental Information 7 Pigment contents Pigment contents were analyzed using SPSS Statistics.

10.7717/peerj.14834/supp-8 Supplemental Information 8 Photosynthetic rate, conductance and soluble sugar Analyzed using SPSS Statistics.

10.7717/peerj.14834/supp-9 Supplemental Information 9 Raw data: consequence of spectrophotometer

10.7717/peerj.14834/supp-10 Supplemental Information 10 Analysis consequence of spectrophotometer measured data

## Additional Information and Declarations

### Data Availability

The following information was supplied regarding data availability: The raw data is available in the Supplemental Files.
